# High Prolactin and Anemia as Factors in Female Infertility: A Cross-Sectional Study in Infertile Women of Balochistan, Pakistan

**DOI:** 10.7759/cureus.84368

**Published:** 2025-05-18

**Authors:** Yasmeen Gul, Noman Sadiq, Somia Iqbal, Muhammad Tahir, Hazar Khan, Mukhtiar Ahmed

**Affiliations:** 1 Obstetrics and Gynecology, Mekran Medical College, Turbat, PAK; 2 Physiology, Mekran Medical College, Turbat, PAK; 3 Physiology, Wah Medical College, Wah, PAK; 4 Pharmacology and Therapeutics, Mekran Medical College, Turbat, PAK; 5 Pharmacology, Mekran Medical College, Turbat, PAK; 6 Pathology, Jhalawan Medical College, Khuzdar, PAK

**Keywords:** anemia, female infertility, female reproductive health, hyperprolactinemia, serum prolactin

## Abstract

Background

Female infertility is a significant health challenge, with hormonal imbalances and nutritional deficiencies being among the key contributing factors. Both hyperprolactinemia and anemia have been suggested as potential factors affecting fertility in women.

Objective

This study aims to determine the prolactin levels and anemia prevalence in infertile women of Balochistan, Pakistan, and to establish whether female infertility is related to hyperprolactinemia and anemia, in addition to the association between hyperprolactinemia and anemia in infertile women.

Methods

This cross-sectional research was conducted in the Department of Obstetrics and Gynecology at Mekran Medical College, Turbat, Balochistan, from January 2024 to June 2024. A total of 310 women diagnosed with infertility were divided into two groups: 222 women with primary infertility and 88 women with secondary infertility. The study participants' prolactin levels were measured, and the prevalence of anemia was determined by measuring hemoglobin levels.

Results

Of the primary infertile women, 148 (47.7%) participants had hyperprolactinemia, whereas 38 (12.3%) secondary infertile women had hyperprolactinemia. The mean serum prolactin levels were significantly higher in primary infertility patients compared to secondary infertility patients (31.85 ± 29.46 ng/mL versus 22.97 ± 18.24 ng/mL, p = 0.009). A p-value of <0.05 (p = 0.001) showed a statistically significant association between infertility type and prolactin levels, with primary infertility showing a higher prevalence of raised prolactin compared to secondary infertility. Mean hemoglobin levels were significantly lower in primary infertility patients (11.58 ± 1.53 g/dL versus 12.14 ± 1.48 g/dL, p = 0.019), with 113 (50.9%) of primary infertile women having anemia compared to 54 (61.4%) of secondary infertile women. In total, 167 (53.8%) infertile women had anemia. Secondary infertility showed a higher prevalence of anemia than primary infertility. However, no statistically significant association existed between prolactin levels and anemia in different infertility types.

Conclusion

Our study shows that the high occurrence of anemia and increased prolactin levels in women of reproductive age suggest their contributory role in causing infertility. Regular screening and prompt intervention for anemia and hyperprolactinemia are critical in treating infertility-related outcomes.

## Introduction

Infertility is defined as the inability to achieve pregnancy after 12 months of regular unprotected intercourse. Of the worldwide adult population, 17.5% suffers from infertility. Primary infertility refers to the inability to conceive or carry a pregnancy to term when a person or couple has never been pregnant before, despite having regular unprotected intercourse for at least 12 months (or six months for women over 35). Secondary infertility, on the other hand, is described as the inability to conceive or maintain a pregnancy after having previously achieved one or more successful pregnancies [1]. In Pakistan, the prevalence of infertility is higher than the global average, recording 18.67%, highlighting infertility as a significant public health issue [2].

The causes of female infertility are multifactorial, but one of the main reasons is hormonal imbalance [3]. Many studies have reported a significant association between hormonal imbalance and female infertility [3,4].

Generally, lactogenic hormones play an essential role in regulating reproductive function [5]. Prolactin, one of the lactogenic hormones, is primarily linked to lactation induction [6]. However, prolactin also has specific receptors in the gonads and plays a significant role in female reproductive physiology [7].

On the one hand, prolactin maintains a regular estrus cycle by stimulating the production of ovarian progesterone, which is essential for the preparation of embryo implantation [8]. On the other hand, hyperprolactinemia is linked with anovulation and can cause infertility. Hyperprolactinemia is defined as serum prolactin levels exceeding 25 ng/mL. Hyperprolactinemia affects approximately 1%-5% of women in the general population, while its prevalence among infertile women ranges from 15% to 30% depending on the population studied [9]. Increased levels of prolactin can be the reason for infertility by elevating hypothalamic dopamine release, which in turn inhibits gonadotropin-releasing hormone (GnRH) and prevents gonadal steroidogenesis and ultimately infertility [10]. Hyperprolactinemia also causes the suppression of kisspeptin neurons that cause GnRH inhibition. In addition, the direct action of prolactin on ovaries causes the inhibition of the human chorionic gonadotropin (hCG)-induced follicle rupture, ensuing ovulation inhibition [8]. Elevated prolactin may be primarily due to normal physiological alterations or pathologically due to diseases affecting the hypogonadal axis, liver, kidneys, and thyroid [11].

Elevated prolactin levels can lead to disturbances in the menstrual cycle and are often linked with heavy or irregular menstrual bleeding, which can be one of the reasons for causing iron deficiency anemia over time [12]. As per the World Health Organization (WHO) reports, the global prevalence of anemia in women of reproductive age is 30%, and in Pakistan, it is 41.3% [13,14]. In iron deficiency anemia, low iron levels impair ovarian function by disturbing follicular development, leading to infertility. In iron deficiency, follicular development gets blocked [15]. Thus, iron is crucial for healthy ovarian function and fertility through adequate follicle development [15].

Several studies show high prolactin levels in infertile women [16]. However, few studies show the association between high prolactin levels and anemia but not in infertile women [17]. Thus, our research aims to assess prolactin levels in infertile women of Balochistan, Pakistan, and to establish whether female infertility is associated with elevated prolactin levels and also a possible link between hyperprolactinemia and anemia in infertile women.

## Materials and methods

Study design and ethical approval

This cross-sectional study was conducted among infertile women after gaining approval from the Ethical Review Committee of Mekran Medical College (approval number: MMC/ERC/18/12/2023). This study was conducted in the Department of Obstetrics and Gynecology at Mekran Medical College, Turbat, Balochistan, from January 2024 to June 2024.

Inclusion and exclusion criteria

Women of reproductive age who were diagnosed with infertility between 18 and 45 years and were attending the infertility clinic for treatment or evaluation were included in the study. The participants were recruited using consecutive sampling of eligible women who visited the infertility clinic during the study period.

The participants with a history of diabetes mellitus, thyroid, or renal diseases that can affect prolactin or anemia status were excluded. The participants who were on medications that affect prolactin (antipsychotic drugs and dopamine agonists) or anemia (iron supplementations) were also excluded from the study. The participants with a history of head trauma or pituitary surgery and with a history of smoking or alcohol consumption were not included in the study. Women with infertility caused solely by male factors were also excluded from the study.

Assessment of parameters

This study intended to investigate the connection between prolactin levels, anemia prevalence, and primary and secondary infertility status in women. The study involved comparing prolactin levels and the presence (hemoglobin level of less than 12.0 g/dL) or absence of anemia between the two infertility groups [18].

Our study involved 310 women diagnosed with infertility. One group was the primary infertility group, with 222 women diagnosed with primary infertility, and the other group was the secondary infertility group, with 88 women diagnosed with secondary infertility.

Prolactin levels were measured in all participants. Serum prolactin was measured using electrochemiluminescence immunoassay (ECLIA) on the Roche Cobas e411 (Basel, Switzerland) analyzer. Blood samples were collected between 8 and 10 AM after overnight fasting to minimize diurnal variation effects. Anemia prevalence was determined by measuring hemoglobin levels in g/dL in all participants.

Data analysis

Data was evaluated using IBM SPSS Statistics for Windows, version 26 (IBM Corp., Armonk, NY). Descriptive statistics were used to calculate the levels of prolactin and the prevalence of anemia among infertile women. Results were displayed in the form of percentages. The chi-square test was used to determine whether there is a noteworthy association between infertility type (primary or secondary) and prolactin levels, and an independent sample t-test was used to measure the difference in prolactin levels between primary and secondary infertility groups. Any p-value of less than the alpha level is deemed statistically significant.

## Results

The sociodemographic characteristics of the study participants are presented in Table 1. A total of 310 women diagnosed with infertility participated in the study, with 222 (71.6%) having primary infertility and 88 (28.4%) having secondary infertility.

**Table 1:** Sociodemographic Characteristics of the Study Participants (N = 310). Data presented as N (%).

Characteristic	Primary Infertility (N = 222)	Secondary Infertility (N = 88)	Total (N = 310)
Age group (years)			
18-25	137 (44.2%)	26 (8.4%)	163 (52.6%)
26-35	79 (25.5%)	57 (18.4%)	136 (43.9%)
36-45	6 (1.9%)	5 (1.6%)	11 (3.5%)
Education status			
No formal education/primary education	154 (49.7%)	66 (21.3%)	220 (71.0%)
Secondary/higher education	68 (21.9%)	22 (7.1%)	90 (29.0%)
Residence area			
Rural	77 (24.8%)	38 (12.3%)	115 (37.1%)
Urban	145 (46.8%)	50 (16.1%)	195 (62.9%)
Family type			
Joint	19 (6.1%)	16 (5.2%)	35 (11.3%)
Nuclear	203 (65.5%)	72 (23.2%)	275 (88.7%)
Duration of marriage (years)			
1-5	108 (34.8%)	62 (20.0%)	170 (54.8%)
6-10	67 (21.6%)	12 (3.9%)	79 (25.5%)
>10	47 (15.2%)	14 (4.5%)	61 (19.7%)

Table 1 shows the sociodemographic characteristics of the study participants. The majority of patients in both groups were within the age range of 18-35 years, had no formal or primary education, lived in urban areas, and belonged to nuclear families.

Table 2 shows the prevalence of anemia in primary infertile (n = 222) and secondary infertile (n = 88) female participants. Results after applying descriptive analysis showed that 113 (50.9%) primary infertile women had anemia, whereas 54 (61.3%) secondary infertile women had anemia. In total, 167 (53.8%) infertile women had anemia.

**Table 2:** Association of Anemia With Primary and Secondary Infertility. Data presented as N (%). The chi-square test was applied. Anemia absent: hemoglobin > 12 g/dL. Anemia present: hemoglobin < 12 g/dL. CI: confidence interval

Fertility Type	Anemia Absent	Anemia Present	Chi-Square (χ²)	Odds Ratio (95% CI)	P-value
Primary infertility	109 (49.1%)	113 (50.9%)	2.69	1.51 (0.92-2.48)	0.101
Secondary infertility	34 (38.6%)	54 (61.4%)			

The result of the chi-square test to determine the association between infertility type (primary or secondary) and anemia status is expressed in Table 2. Results after applying the chi-square test and the odds ratio of 1.51 (95% confidence interval {CI}: 0.92-2.48) show that women with secondary infertility have 1.51 times higher odds of having anemia compared to women with primary infertility.

Table 3 compares prolactin levels and blood parameters between primary and secondary infertility groups. Mean serum prolactin levels were significantly higher in primary infertility patients compared to secondary infertility patients (31.85 ± 29.46 ng/mL versus 22.97 ± 18.24 ng/mL, p = 0.009). Hemoglobin levels also showed a statistically significant difference (p = 0.019), with secondary infertility patients having higher mean hemoglobin levels compared to primary infertility patients (mean difference, 0.56 g/dL; 95% CI, 0.09-1.03). No significant differences were found in other blood parameters between the two groups. The analysis of other hematological parameters including red blood cells (RBC), white blood cells (WBC), hematocrit, mean corpuscular volume (MCV), mean corpuscular hemoglobin (MCH), mean corpuscular hemoglobin concentration (MCHC), and platelets showed no statistically significant differences between primary and secondary infertility groups (all p > 0.05), suggesting that these parameters may not play a distinctive role in differentiating between the two infertility types in our study population. No significant association was found between prolactin level and anemia.

**Table 3:** Comparison of Prolactin Levels to Complete Blood Count Parameters Between Primary and Secondary Infertility Participants. Data presented as mean ± standard deviation (SD). An independent sample t-test was applied. *Statistically significant (p < 0.05). RBC, red blood cells; WBC, white blood cells; MCV, mean corpuscular volume; MCH, mean corpuscular hemoglobin; MCHC, mean corpuscular hemoglobin concentration

Parameter	Primary Infertility (N = 222)	Secondary Infertility (N = 88)	T-statistic	P-value
Prolactin (ng/mL)	31.85 ± 29.46	22.97 ± 18.24	2.636	0.009*
Hemoglobin (g/dL)	11.58 ± 1.53	12.14 ± 1.48	2.367	0.019*
RBC (×10^6^/μL)	4.72 ± 0.61	4.81 ± 0.58	1.194	0.234
WBC (×10^3^/μL)	8.26 ± 2.31	8.18 ± 2.24	0.276	0.783
Hematocrit (%)	37.09 ± 4.35	36.87 ± 4.62	0.397	0.692
MCV (fL)	77.52 ± 13.87	73.98 ± 16.84	1.878	0.061
MCH (pg)	25.57 ± 4.39	25.62 ± 4.35	0.087	0.931
MCHC (g/dL)	31.24 ± 4.47	31.63 ± 3.39	0.764	0.446
Platelets (×10^3^/μL)	296.86 ± 70.34	290.28 ± 92.41	0.671	0.503

## Discussion

Female infertility is a complex condition with multifactorial etiology. Our study was conducted to investigate the relationship between prolactin levels, anemia, and infertility status in women from Balochistan, Pakistan. The findings reveal important associations contributing to our understanding of infertility in this region.

Our study demonstrates a significant relationship between hyperprolactinemia and primary infertility, revealing that women with primary infertility have substantially higher prolactin levels compared to those with secondary infertility. These findings align with previous research that observed elevated prolactin levels in infertile women compared to fertile controls [19]. The pathophysiological mechanism underlying this association can be explained by the inhibitory effect of hyperprolactinemia on the hypothalamic-pituitary-gonadal axis. When prolactin levels rise abnormally, they cause a decrease in estrogen and progesterone production, leading to aberrant follicular development and ultimately resulting in anovulation and infertility [20].

Our observation that primary infertile women have a higher prevalence of elevated prolactin levels compared to women with secondary infertility corroborates findings from recent research in South Punjab, Pakistan [16]. This difference can be attributed to the mechanism by which hyperprolactinemia inhibits endogenous gonadotropins, resulting in anovulatory cycles that prevent conception entirely. In women who have previously conceived (secondary infertility), other factors might play a more predominant role in their current infertility status. At the same time, prolactin dysregulation appears to be a more critical factor in primary infertility cases.

The high occurrence of anemia among our study population is particularly concerning and aligns with several regional studies from Pakistan and other low- and middle-income countries [21-23]. This high prevalence can be attributed to multiple factors, including poor nutritional status, lower socioeconomic conditions, the lack of awareness about iron-rich diets, and menstrual dysfunctions that are common in Pakistan. The significantly higher prevalence of anemia in primary infertile women as compared to secondary infertile women in our study suggests that iron deficiency may play a more crucial role in the pathogenesis of primary infertility.

The mechanism by which anemia contributes to infertility has been elucidated in recent research demonstrating that iron deficiency induces the failure of follicular development in mice [15]. This animal model provides valuable insights into how chronic iron deficiency might impair human ovarian function. Iron is essential for various cellular processes, including DNA synthesis, enzyme activity, and cellular respiration, which are crucial for proper follicular development and oocyte maturation. When iron levels are insufficient, these processes are compromised, potentially leading to poor oocyte quality, impaired follicular development, and infertility.

While our study did not find a statistically significant association between prolactin levels and anemia in different infertility types, this finding warrants further investigation. The relationship between prolactin and iron metabolism is complex and not fully understood. However, research has reported that high prolactin is one of the principal causes of menstrual disturbances, which can subsequently lead to iron deficiency anemia due to excessive menstrual blood loss [24]. Additionally, both hyperprolactinemia and anemia can independently affect the hypothalamic-pituitary-gonadal axis, potentially creating a synergistic effect on fertility even without a direct correlation between the two conditions. Moreover, the lack of significant association between prolactin levels and anemia may be influenced by the severity and duration of hyperprolactinemia, which were not assessed in our cross-sectional design. Other hormonal factors that were not measured (such as estrogen and progesterone) might mediate or modify this relationship, or the multifactorial nature of anemia in this population could dilute any direct association with prolactin levels.

The implications of our findings extend beyond just understanding the epidemiology of these conditions. They highlight the importance of routine screening for both prolactin levels and anemia in women presenting with infertility, especially in resource-limited settings such as Balochistan, Pakistan. The early detection and management of these conditions could potentially improve fertility outcomes and reduce the psychological and social burden associated with infertility.

Our study has several strengths, including a robust sample size and clear differentiation between primary and secondary infertility cases. However, some limitations should be acknowledged. The cross-sectional design prevents us from establishing causality, and excluding various comorbidities, while necessary for methodological reasons, may limit the generalizability of our findings. Additionally, we did not assess other hormonal parameters such as thyroid function tests and follicle-stimulating hormone (FSH), luteinizing hormone (LH), and estradiol levels, which could provide a more comprehensive understanding of the hormonal milieu in these infertile women.

Future research should focus on longitudinal studies to establish causality and interventional studies to determine whether correcting hyperprolactinemia and anemia improves fertility outcomes. Additionally, exploring the potential molecular interactions between iron metabolism and prolactin regulation could provide valuable insights into the pathophysiology of infertility.

## Conclusions

Our study highlights that both hyperprolactinemia and anemia are highly prevalent among infertile women in Balochistan, Pakistan. Our findings demonstrate significant differences between primary and secondary infertility groups, with primary infertility showing higher hyperprolactinemia and secondary infertility showing higher anemia, suggesting that distinct pathophysiological mechanisms may be at play in these two types of infertility. The increased occurrence of these conditions among women of reproductive age suggests their potential contributory role in causing infertility. Given these findings, regular screening and prompt intervention for anemia and hyperprolactinemia should be considered as essential components in the management of female infertility. Healthcare providers should incorporate routine hemoglobin and prolactin measurements in their evaluation of infertile women, especially in regions with high prevalence of these conditions. The early detection and appropriate management of these conditions could potentially improve fertility outcomes and reduce the burden of infertility in the population.
